# Development of sol–gel derived fluoridated hydroxyapatite/CaTiO_3_ composite coating on titanium implant

**DOI:** 10.1007/s10856-026-07142-y

**Published:** 2026-11-13

**Authors:** Alaa Jaafar, Christian Schimpf, Marcel Mandel, Hans Lesny, Christine Hecker, David Rafaja, Lutz Krüger, Pal Arki, Yvonne Joseph

**Affiliations:** 1 Institute of Nanoscale and Biobased Materials, TU Bergakademie Freiberg, Freiberg, Germany; 2 Center for Efficient High Temperature Processes and Materials Conversion, TU Bergakademie Freiberg, Freiberg, Germany; 3 Institute of Materials Science, TU Bergakademie Freiberg, Freiberg, Germany; 4 Institute of Materials Engineering, TU Bergakademie Freiberg, Freiberg, Germany

## Abstract

The difficulties in regulating the degradation of hydroxyapatite (HA)-coated titanium implants necessitate the development of innovative solutions to ensure their biological safety and long-term durability. This study aims to investigate the combined effects of CaTiO₃ incorporation and controlled fluoride substitution on HA-based composite coatings deposited onto Ti6Al4V substrates via sol–gel, with fluoridation levels systematically varied to assess their structural, mechanical, chemical, and biological performance. The phase composition, chemical bonding, elemental composition, and coating morphology were characterized by XRD, FTIR, XPS, and SEM, respectively. The composite coatings’ modulus of elasticity and hardness were investigated using a nanoindentation test, which has shown that CaTiO_3_ and fluoride ions considerably improve the mechanical properties of the HA layers. Moreover, the adhesion strength of the composite coatings has shown significant improvement with increasing fluoridation degree. The investigation of the dissolution behavior showed that all fluoridated apatite coatings have a lower degradation rate, which indicates the possibility of tailoring the solubility of apatite structures in phosphate-buffered saline (PBS) as fluoride is incorporated. The potentiodynamic polarization test demonstrated that composite coatings on titanium alloy exhibit a lower corrosion rate than pure HA-coated samples. The fluoride-doped coatings exhibited antibacterial efficacy against *S. aureus and E. coli* bacteria. In vitro cellular responses evaluated with human osteosarcoma MG-63 cells revealed stimulated cell proliferation and differentiation activities as fluoride was incorporated. This study demonstrates that fluoridated apatite/CaTiO_3_ composite coatings have several advantages over pure hydroxyapatite coatings in terms of mechanical properties, chemical stability, and cellular responses.

**Figure:**
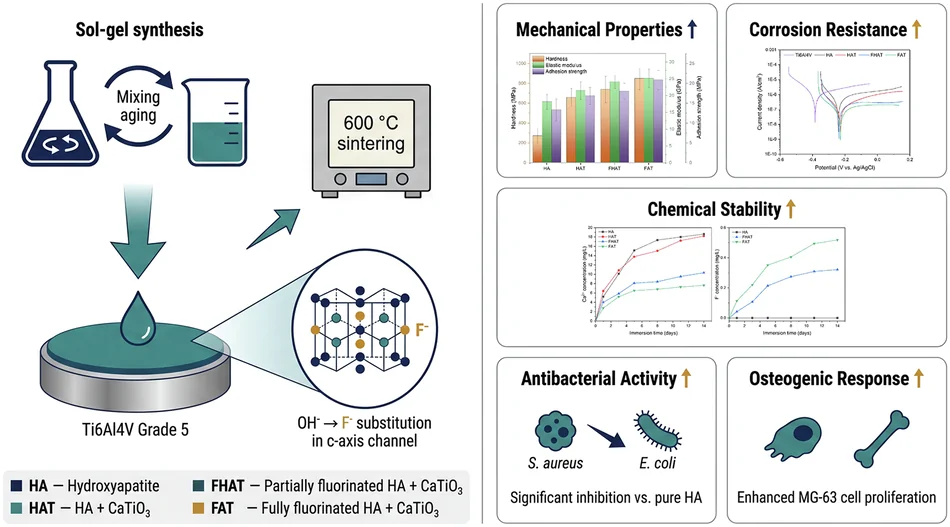

## Introduction

Surface properties of metallic implants are considered one of the essential features controlling their durability and functionality. The scientific community has paid much attention to improving the surface properties of implants with respect to their mechanical properties and chemical environments, where these materials are intended to be employed [1]. In orthopedics, grade 5 of titanium alloy (Ti6Al4V) has been widely used owing to its unique combination of physical, mechanical, and chemical properties. Nevertheless, the concerns related to the release of Al and V ions from these materials and their bio-inertness have motivated the modification of their surfaces with bioactive coatings. This modification was proven to be a practical approach to retain appropriate mechanical performance, high biocompatibility, and bioactivity of the surface [2, 3].

Hydroxyapatite (HA, Ca10(PO4)6(OH)2) is one of the most interesting bioactive ceramics that have been extensively considered as a coating for Ti implants due to its high biocompatibility and osteoinductivity [4–7]. The modification of implant surfaces with this material improves the biological fixation at the bone-implant interface due to its ability to induce bone-like apatite formation. However, the poor mechanical and chemical properties of HA coatings, such as low fracture toughness, poor adhesion to the substrate material, and high biodegradation in the body environment, affect their long-term reliability [8, 9]. Therefore, many efforts have been undertaken to enhance the chemical stability and mechanical performance of the HA coatings and to improve their adhesion to the substrate [9]. Among different approaches, the reinforcement by ceramic particles is considered a reliable solution to overcome the limitations of pure HA coatings [4]. In this context, titanium dioxide (TiO2) has drawn much attention owing to its chemical affinity toward HA coating and Ti6Al4V substrate, high biocompatibility, and its ability to enhance the mechanical strength of HA coatings [4]. Many studies have illustrated the vital role of TiO2 reinforcement in improving the surface characteristics, corrosion resistance, and adhesion of HA coatings [1, 9–13]. As an alternative to TiO₂, calcium titanate (CaTiO₃) has been reported as a beneficial Ti-containing phase in HA-based coatings. Unlike TiO₂, which typically remains as a discrete secondary phase, CaTiO₃ forms through solid-state reactions between Ti-containing species and the HA matrix during thermal treatment [14] and is biocompatible and chemically stable in physiological environments, forming strong interfaces with both the apatite matrix and the Ti substrate, which contributes to improved mechanical performance and fixation [15]. The favorable in vivo response observed for CaTiO₃-coated implants [16, 17] suggests that incorporating CaTiO₃ in HA coatings can improve the interfacial stability and biological performance. In vitro, CaTiO₃-containing coatings have been reported to enhance osteoblast adhesion, increase alkaline phosphatase (ALP) activity, and support osteogenic differentiation compared to unmodified titanium or HA surfaces [18, 19]. These data indicate that CaTiO₃ can act as an effective Ti-bearing reinforcement phase for HA, combining the bioactivity of apatite with additional mechanical and biological advantages.

On the other hand, incorporating fluoride into the HA structure is a well-established approach for enhancing the structural stability, biological activity, and mechanical performance. Fluoride ions (F-) are essential trace elements in human bone that promote dissolution resistance. Doping fluoride ions in the HA structure is characterized by a higher binding energy than that of the hydroxyl groups [20]. In addition, in vivo investigations in a human trial revealed that fluoridated HA possesses a better biological performance than the conventional HA [21, 22]. Furthermore, fluoride ions can accelerate the crystallization and mineralization of hydroxyapatite in bone formation and reduce bacterial activity [23–25]. There is also growing evidence that fluoride ions stimulate cell proliferation and slightly increase ALP activity [26–30]. However, several studies have reported the potentially toxic effects of fluoride ions when excessively released, as they noticed low proliferation rates of osteoblast cells [31]. Therefore, well-designed fluoridated apatite coatings with precise control of the amount of released fluoride ions are essential to achieve biological, structural, and mechanical improvements while retaining comparable bioactivity and biocompatibility to undoped HA coatings in terms of tissue and cell responses.

Numerous coating methods have been adopted for HA deposition on titanium substrates, such as sol–gel coating [32–36], plasma spraying [37–39], biomimetic deposition [40], electrochemical deposition [41, 42], and electrophoretic deposition [43, 44], etc. Among these, sol–gel coating has several advantages over other techniques, as it is a simple and cheap preparation method that provides high purity and homogeneity at low processing temperatures [45].

Although CaTiO₃-reinforced HA coatings and fluoridated HA coatings have been individually reported in the literature, published information on the simultaneous effects of fluoride substitution and the presence of CaTiO₃ as a reinforcing phase within a single HA-based coating system remains scarce. In particular, the combined influence of these two modifications on mechanical integrity, dissolution behavior, corrosion resistance, and biological performance has not been systematically characterized. Therefore, this study aims to modify the Ti6Al4V alloy surface by depositing fluoridated HA/CaTiO_3_ composite coatings using sol–gel and comprehensively investigate their structural, mechanical, chemical, and biological properties. The hydroxyapatite/CaTiO₃, partially fluoridated hydroxyapatite/CaTiO₃, and fully fluoridated hydroxyapatite/CaTiO₃ coatings are coded as HAT, FHAT, and FAT, respectively. The structural properties of the composites were characterized using X-ray diffraction analysis (XRD), Fourier-transform infrared spectroscopy (FTIR), X-ray photoelectron spectroscopy (XPS), and scanning electron microscopy (SEM). Pull-out and nanoindentation tests were performed to characterize the mechanical properties, including bonding strength, hardness, and elastic modulus. Chemical stability of the coatings, including corrosion behavior and ion release, was assessed through potentiodynamic polarization testing in simulated body fluid (SBF) and through the analysis of released ions using a fluoride-ion-selective electrode and inductively coupled plasma–atomic emission spectrometry (ICP-AES). The biological performance of the composites was evaluated via antibacterial activity, cell proliferation, and differentiation.

## Methodology

### Sample preparation

Titanium alloy samples (Ti6Al4V, Grade 5, obtained from Horst zu Jeddeloh, Germany), with 5 mm in thickness and 15 mm in diameter, were wet-ground with silicon carbide emery papers to eliminate contamination and macro flaws. The ground samples were cleaned using ultrasonic waves for 15 min with ethanol, acetone, and deionized (DI) water.

The HA sol was prepared according to the recommended synthesis route outlined in a previous study [46]. Briefly, deionized (DI) water was mixed with 2 M triethyl phosphite ((C2H5O)3 P, 98% pure, Alfa Aesar) diluted in ethanol for 24 h with an H2O/(C2H5O)3 P ratio equal to 4. Calcium nitrate (Ca(NO3)2*4H2O; 99% pure, Merck) was hydrolyzed in ethanol for 24 h to prepare a 2 M solution, and added dropwise to the hydrolyzed phosphorus solution in stoichiometric amount (Ca/P ∼1.67). The pH of the resulting mixture was adjusted by adding 5 vol.% of NH4OH (32%, Merck). After agitation for 3 h, the sol was aged in static mode for 72 h. The fluoridated apatite sols were prepared by adding ammonium fluoride (NH4F, 99% pure, Merck) to the phosphorus solutions in different amounts. The P/F molar ratio was set at 6 and 3 to achieve the corresponding compositions of fluorhydroxyapatite (FHA, Ca10(PO4)6F1OH1) and fluorapatite (FA, Ca10(PO4)6F2) by substituting the hydroxyl group with fluoride ions in 1 and 2 molar ratios, respectively. After stirring for 24 h, the hydrolyzed calcium solutions were added dropwise to the P + F solutions, maintaining the Ca/P ratio close to the stoichiometric value. Subsequently, the protocol developed for preparing pure HA sol was followed [46].

For the preparation of the TiO2, tetrabutylorthotitanate (Ti(OCH2CH2CH2CH3)4; 98%, Merck) and diethanolamine (HN(CH2CH2OH)2; 99.5%, Merck) were mixed with ethanol. The addition of ethanol might cause a rapid precipitation of insoluble titanium oxy-hydroxide/hydrated titania due to the high tetrabutylorthotitanate reactivity. Thus, in order to stabilize the hydrolysis process, diethanolamine was first mixed with ethanol, and then tetrabutylorthotitanate was added. Ethanol and water were added dropwise after 2 h of active stirring. After agitation for 2 h, the solution was aged for 24 h. The components’ molar ratios were as follows: Ti(OC_4_H_9_)_4_:C_2_H_5_OH: NH(C_2_H_4_OH)_2_:H_2_O = 1:26.5:1:1 [47]. The composite sols were prepared by mixing 30 mol. % of Ti-based sol gradually with HA, FHA, and FA sols for 16 h to obtain HAT, FHAT, and FAT, respectively. The molar percentage of the Ti-containing additive was selected based on the recommendations of previous studies [12, 45, 48].

After the sols were successfully prepared via sol–gel reactions, part of these sols was used to prepare powder samples by drying for 12 h at 80 °C. The dried gel was then crushed into a fine powder and heated for 1 h at 600 °C. Prepared sols were deposited on polished Ti6Al4V substrates via spin coating at a rotational speed of 2500 rpm and a spinning duration of 15 s. Subsequently, the coated samples were dried for 12 h at 80 °C and sintered for 1 h at 600 °C. The coating process was repeated twice to achieve the desired thickness.

### Materials characterization

The phase composition, lattice parameters, crystallite size, and microstrain of all crystalline phases were determined by X-ray diffraction (XRD) and subsequent full powder pattern refinement using the MAUD software package [49]. The XRD patterns were measured on a SEIFERT-FPM URD6 diffractometer (Freiberger Präzisionsmechanik GmbH | Rich. Seifert Co. GmbH & Co. KG, Germany), which was equipped with a sealed X-ray tube producing Cu-Kα radiation (□_1_ = 1.54056 Å, □_2_ = 1.54439 Å). For the XRD analysis, pulverized samples were distributed evenly on a Si single-crystal zero-background holder. The diffracted beam passed a graphite monochromator that suppressed unwanted radiation components before it was detected by a scintillation counter. The XRD data were collected over 2θ ranging from 20° to 80° with a step size of 0.02° and with a dwell time of 12 s. For the analysis of the crystallite size and microstrain, the instrumental function of the diffractometer was calibrated by a LaB_6_ standard (NIST SRM 660b) [50].

The functional groups of the prepared powders were determined with the aid of the Fourier transform infrared spectrometry (Bruker Tensor II) using the KBr pellet method. The FTIR analysis was carried out at room temperature. The IR spectra were acquired in the wavenumber range 400–4000 cm^−1^ with a scanning resolution of 2 cm^−1^. X-ray photoelectron spectrometry (XPS, ESCALAB 250Xi, Thermo Fisher Scientific) with an aluminum (*Al*-*Kα*) excitation source has been used to analyze the chemical composition of the coatings quantitatively. Scanning electron microscopy (SEM, Phenom G2, Thermo Scientific) was utilized to investigate the surface morphology of the coatings.

Contact angle measurements were conducted to evaluate the hydrophilicity of coated samples to SBF at 37 °C using a Krüss DSA25E goniometer. The instructions of Kukubo and Takadama [51] were adopted to prepare the SBF solution with ion concentrations that resemble those of human blood plasma. The average of five independent measurements of each specimen, with the standard deviation, was determined.

Nanoindentation tests have been applied to assess the mechanical properties of the developed coatings, including modulus of elasticity and hardness [52]. The test was carried out using a Hysitron TriboScope nano-mechanical testing system equipped with a three-sided pyramidal-shaped indenter at a peak load of 5 mN. Multiple indentations at different spots of the coatings were performed, and the results were computed using the Oliver-Pharr method [53].

The adhesion strength of the applied coatings was assessed by a pull-out test based on ASTM-C633 recommendations [54]. Epoxy resin was used to glue the coated samples to two titanium rods, then cured at 150 °C for 1 hr. A conventional tensile test was carried out at a constant load speed of 1 mm/sec using a universal testing machine (WDW-200KN, USA). The adhesion strength represented the maximum tensile stress necessary to peel off the coating layer.

### Dissolution behavior analysis and corrosion investigation

The dissolution behavior of the coated samples was assessed by immersing them in PBS at 37 °C for different periods of up to 14 days. At each period, the coated samples were taken out from the solution, and the PBS solution was analyzed to detect the concentration of fluoride and calcium ions released from the coating using a fluoride-ion selective electrode and inductively coupled plasma–atomic emission spectrometry (ICP-AES, ICPS-100IV, Shimadzu, Japan), respectively.

The electrochemical corrosion behavior of the prepared coated samples has been tested by potentiodynamic polarization test at 37 °C in SBF using a potentiostat (VSP, Bio-Logic Science Instruments). Firstly, the open-circuit potential (OCP) was measured for 30 min for each sample, ensuring steady-state conditions. Subsequently, the polarization test was performed using a three-electrode cell configuration, with a platinum electrode serving as a counter electrode, the coated Ti6Al4V alloy as the working electrode, and a saturated silver-silver chloride solution as the reference electrode. The polarization test was carried out in a potential range of ±250 mV vs. *E*_OCP_ at a scan rate of 0.1 mV/s. Tafel extrapolation method extracted the corrosion parameters, including corrosion potential (E_corr_) and corrosion current density (I_corr_). The corrosion rate (CR) was calculated based on *I*_corr_ values according to Eq. 1 [55]:

1 $${CR}[{{{\rm{mm}}}}/{{{\rm{y}}}}]=22.8[({{{{\rm{mm}}}}* {{{\rm{cm}}}}}^{2})/({{{{\rm{y}}}}* 10}^{-3}{{{\rm{A}}}})]\times {I}_{{{\rm{corr}}}}[{10}^{-3}{{{\rm{A}}}}/{{{{\rm{cm}}}}}^{2}]$$

### Biological properties assessment

#### Antibacterial efficacy

The antibacterial efficacy of the developed coatings was assessed by the plate-counting method against S. aureus and *E. coli* bacteria [56, 57]. Before the bacterial culture test, the coated samples were sterilized in an autoclave at 121 °C for 30 min. Standard laboratory strains of E. coli and S. aureus were used for the antibacterial assay. The bacterial solution was prepared by continuous oscillation in a Luria-Bertani medium for one day. The obtained bacterial solution was diluted in physiological saline to 5 × 10^4^ CFU/mL^–1^ concentration, and 50 μL of bacteria culture was dropped onto the surfaces of the samples and cultured at 37 °C for 24 h in a humidified incubator. To remove the adhering bacteria from the coating surface, 1 mL of physiological saline was dripped on the coated samples and vortexed for 10 min. Then, 20 μL of bacterial suspension was spread uniformly onto Mueller-Hinton agar medium and cultured for 24 h. The antibacterial assay was performed in triplicate, and colony counts are reported as the mean ± standard deviation. The number of surviving bacterial colonies was counted visually and the antibacterial rate (R) was calculated according to Eq. 2:

2 $${{{\rm{R}}}}=(1-{{{\rm{N}}}}/{{{{\rm{N}}}}}_{0})\times 100$$

Where N_0_ is the number of colonies of pure HA coating, and N is the corresponding number of colonies of HAT and fluoridated HAT composite coatings.

#### Cell culture assay

A human osteosarcoma cell line, MG-63, was adopted to assess the biological performance of the composite coatings. Firstly, the MG-63 cells were cultured in a culture medium containing Dulbecco’s modified Eagle’s medium (DMEM) supplemented with 10% fetal bovine serum and 1% penicillin and streptomycin at 37 °C in a 5% CO_2_ humidified incubator. The culture medium was refreshed every two days until reaching 90% confluence. Cells were subcultured under standard conditions and used at low passage numbers to ensure consistent biological behavior. The trypsinization process was performed to detach cultured cells. Then, the cells were resuspended in a culture medium and used for the experiments. Cell assays, including ALP activity and cell proliferation, were carried out to assess the cellular response to the composite coatings.

The proliferation of MG-63 cells was assessed by performing an MTT assay on the prepared samples. The cells at a density of 5 × 10^4^ cells/well were seeded on the coated samples in a 24-well plate and cultured at 37 °C in a 5% CO_2_ humidified incubator for 1, 3, and 5 days. At each period, the coated samples were rinsed with phosphate-buffered saline. Then 25 μL/well of dimethyl thiazolyl diphenyl tetrazolium (MTT, 5 mg/mL) solution was poured into the cell cultures and incubated for 4 h at 37 °C in a humidified atmosphere. Then, the produced formazan crystals were dissolved by adding 100 μL of dimethyl sulfoxide (DMSO; Sigma-Aldrich, St. Louis, MO, USA) for a period of 15 min. Afterward, the optical density of the solved solutions, which is correlated to the viable cell number, was measured using an ELISA reader at 570 nm. All experiments were performed in quadruplicate for each set of samples.

The differentiation of MG-63 cells was evaluated by employing an ALP activity assay on the composite coatings. The MG-63 cells were seeded on each coated specimen in a 24-well plate and then cultured for 7 and 14 days at 37 °C in a humidified atmosphere at a density of 1 × 104 cells/ml. The cell layer was rinsed in PBS and detached with a trypsin-EDTA solution (Gibco, Thermo Fisher Scientific, Waltham, MA, USA) at each period. After centrifugation, the cell pellets were rinsed in phosphate-buffered saline and suspended again in 0.5 ml DI water with 0.1% Triton X-100 solution for cell lysis. After 2 h of incubation, the solution was homogenized by a cyclic freezing/thawing process. 100 µl of cell lysate was mixed with 100 µl of p-nitrophenyl phosphate (pNPP) substrate solution (Sigma-Aldrich, St. Louis, MO, USA) and incubated at 37 °C for 30 min. A Bio-Rad protein assay kit (Bio-Rad Laboratories, Hercules, CA, USA) was employed to measure the content of protein in the cell lysate; then, ALP activity of the cells was measured colorimetrically by detecting the transformation of pNPP substrate into p-nitrophenol (pNP) after it was mixed in equal volume with the cell lysate and incubated for 30 min. The optical density of the specimens was measured using a microplate spectrophotometer at 405 nm. The calculated ALP activity was normalized by the total protein content and expressed as micromoles of transformed pNPP into pNP per mg of total protein per h. All experiments were performed in quadruplicate for each set of samples.

#### Statistical analysis

Statistical differences between the obtained results data were determined by a one-way analysis of variance followed by the Tukey post hoc multiple comparison test to determine the statistical differences. Statistical significance was considered at *p*-value < 0.05.

## Results

**Fig. 1:**
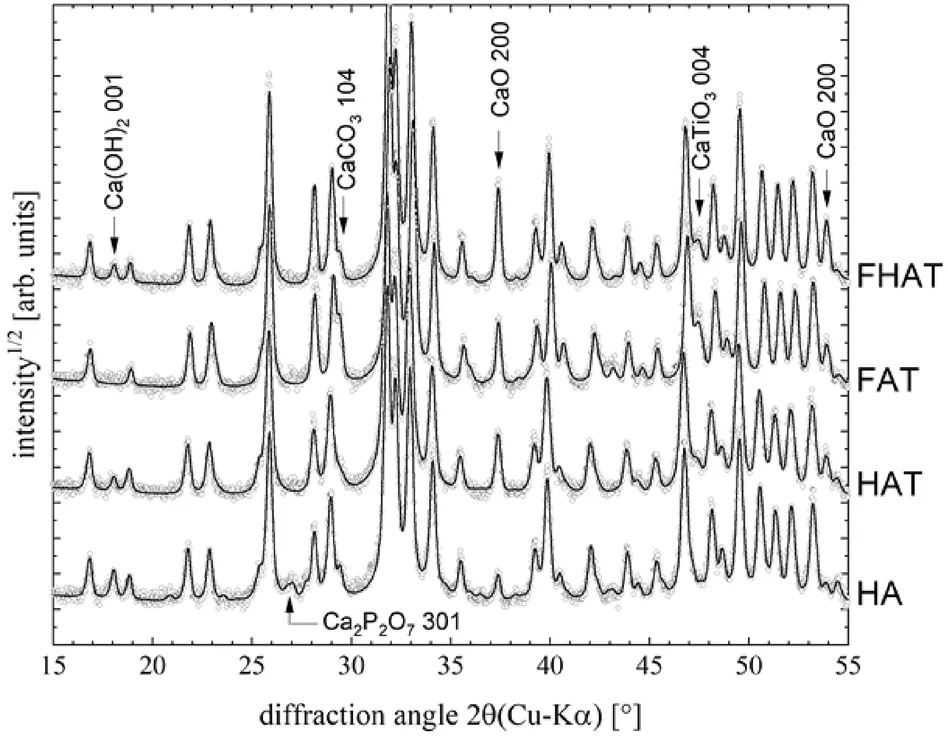
Low-angle sections of powder XRD patterns measured for different apatite samples: HA hydroxyapatite, HAT hydroxyapatite/CaTiO_3_, FAT: fully fluorinated hydroxyapatite (fluorapatite) with CaTiO_3_, and FHAT: partially fluorinated hydroxyapatite/CaTiO_3_. Dots represent measured intensities; solid lines are the results of the Rietveld refinement. Prominent peaks of the minority phases are labelled

Analysis of the XRD patterns of the HA, HAT, FAT and FHAT samples (Fig. 1) confirmed that these samples contain hydroxyapatite, Ca10(PO4)6(OH)2 (ICDD-PDF-5 #01-071-5048), hydroxyfluorapatite, Ca10(PO4)6(OH)2-xFx [58], or fluorapatite, Ca10(PO4)6 F (ICDD-PDF-5 #00-015-0876) as the main component. All compounds crystallize in the same space group (P6₃/m). Consistent with this behavior, a systematic decrease of the lattice parameter a is observed for the fluoridated samples (FHAT and FAT) compared to HAT (Table 1), whereas the c parameter remains almost constant. The crystallite size and microstrain values obtained from the refinements indicate that partial fluoridation (FHAT) is associated with larger apatite crystallites and lower microstrain compared to HAT. A higher degree of fluoridation (FAT) results in a reduction of the crystallite size and a slight increase in the microstrain. Table 1 summarizes the structural parameters of the primary apatite phase obtained from Rietveld refinement.

**Table 1:** Volume fractions, lattice parameters, crystallite sizes, and microstrain values of the primary apatite phase (space group P6_3_/m) in individual samples

Property	HA	HAT	FAT	FHAT
Volume Fraction (vol. %)	93 ± 4	92 ± 4	89 ± 4	92 ± 4
Lattice Parameter *a* (Å)	9.4165 ± 0.0003	9.4227 ± 0.0004	9.3746 ± 0.0004	9.3988 ± 0.0003
Lattice Parameter *c* (Å)	6.8837 ± 0.0003	6.8897 ± 0.0004	6.8831 ± 0.0003	6.8878 ± 0.0003
Crystallite Size (nm)	124 ± 6	105 ± 16	156 ± 13	206 ± 12
Microstrain (10^-4^)	7 ± 1	13 ± 1	9 ± 1	8 ± 1

In addition to the primary apatite phase, minor secondary phases were detected in the apatite samples (summarized in Table 2), including CaO, CaCO₃, Ca(OH)₂, Ca₂P₂O₇, and CaTiO₃. In the samples with the addition of Ti-precursors, which were calcined at 600 °C, CaTiO₃ was identified as the dominant Ti-containing crystalline phase. The absence of any reflections attributable to rutile or anatase TiO₂ suggests that most of the Ti-containing sol is converted into CaTiO₃ during calcination at 600 °C.

**Table 2:** Secondary phases identified in the synthesized powders and their volume fractions

Minor Phase	(ICSD-No.)	Space Group	Volume fraction of secondary phases (%)			
			HA	HAT	FAT	FHAT
CaO	(00-037-1497)	${Fm}\bar{3}m$	∼1	2 ± 0.5	2 ± 0.5	3 ± 0.5
CaCO_3_	(00-005-0586)	$R\bar{3}c$	∼1	2 ± 0.5	4 ± 1	∼1
Ca(OH)_2_	(00-044-1481)	$P\bar{3}m1$	2 ± 0.5	∼1		∼1
Ca_2_P_2_O_7_	(04-009-2362)	$P{2}_{1}/n$	3 ± 0.5			
CaTiO_3_	(01-077-8906)	Pbnm		3 ± 0.5	5 ± 1	3 ± 1

**Fig. 2:**
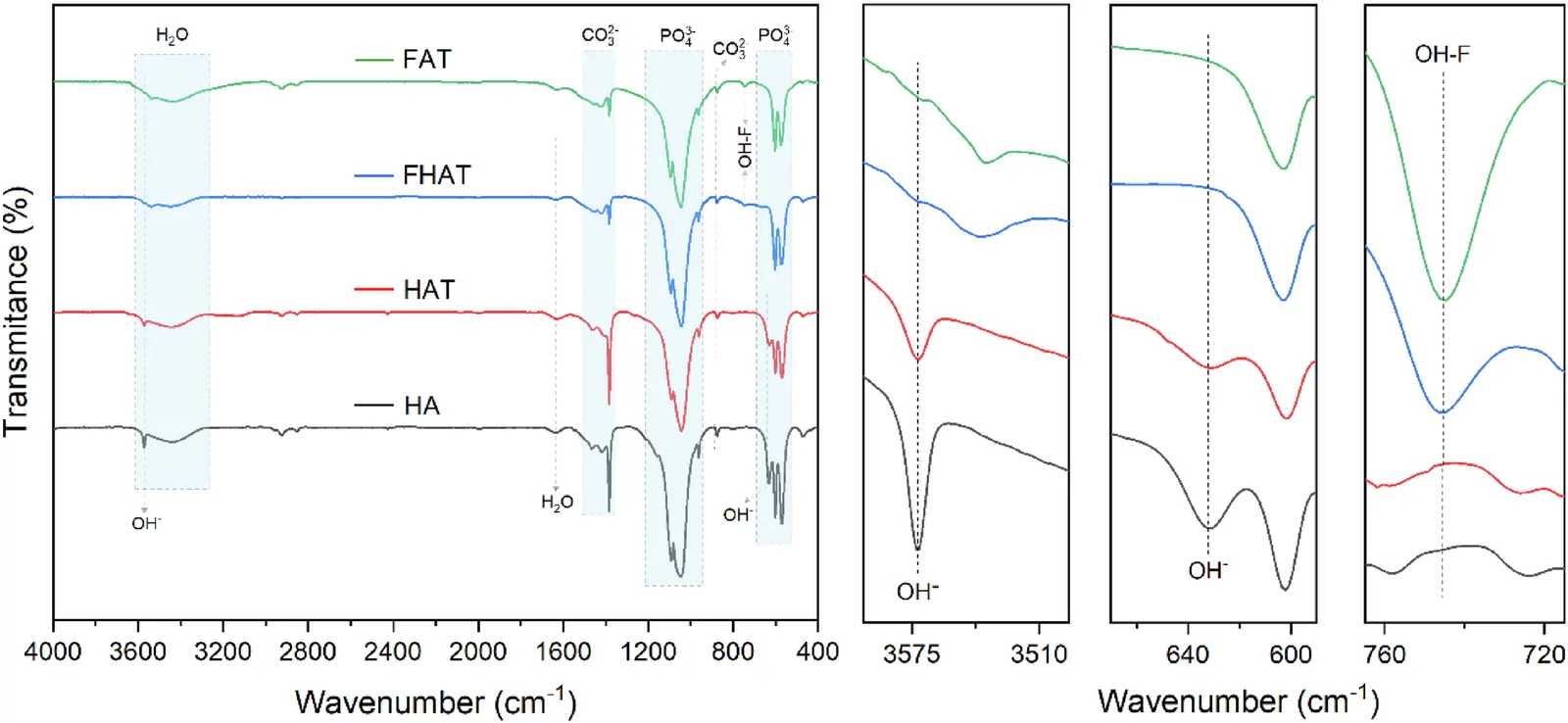
FTIR spectra of prepared powders

The FTIR spectra of the HA and its composites, as comparatively displayed in Fig. 2, represent characteristic bands of PO₄³⁻ and OH⁻ functional groups corresponding to the apatite structure. The characteristic absorption bands at 572 and 602 cm⁻¹ are assigned to the vibrational bending mode of O–P–O linkage in the PO₄³⁻ groups, while the bands at 963, 1047, and 1092 cm⁻¹ are attributed to the vibrational stretching mode of PO₄³⁻ groups [59]. The vibrational modes of OH⁻ groups were observed at 632 and 3573 cm⁻¹, corresponding to the characteristic hydroxyl groups of HA [60]. The peak at 1638 cm⁻¹ and the broad band from 3300 to 3600 cm⁻¹ are related to the vibrational stretching mode of H-O-H groups. The low-intensity peak at 875 cm⁻¹ and the band at 1384 cm⁻¹ are assigned to the vibrational stretching mode of the C–O bond of CO₃²⁻ ions. The FTIR spectrum of the HAT sample closely resembles that of pure HA.

As fluorine was incorporated, the band at 3573 cm⁻¹ was shifted to 3540 cm⁻¹ for FHAT and 3537 cm⁻¹ for FAT and exhibited lower intensity. In addition, the hydroxyl band at 632 cm⁻¹ was not detected in the fluoridated coatings. An additional band at 746 cm⁻¹ was observed in the FHAT and FAT spectra.

**Fig. 3:**
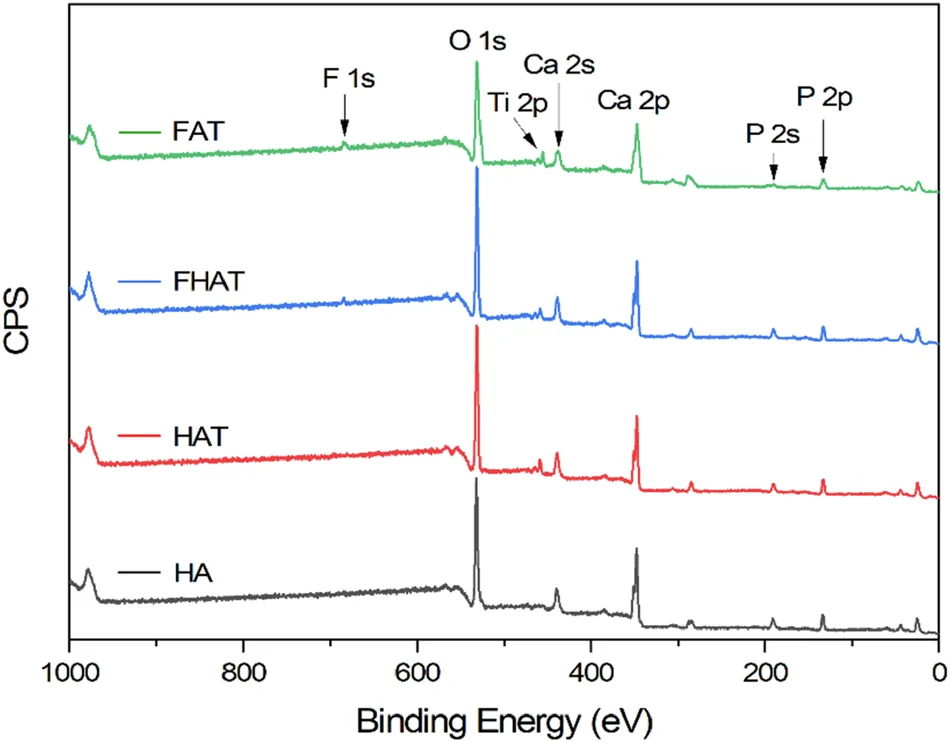
The XPS survey spectra of pure and composite coatings

As illustrated in Fig. 3, the wide energy survey scans of XPS spectra for the HA, HAT, FHAT, and FAT show characteristic signals of oxygen, carbon, calcium, and phosphorus. Representative high-resolution XPS spectra for FAT are provided in Figure S1 (Supporting Information). The high-resolution spectra of Ca 2p exhibited two peaks at ∼347.3 eV and ∼350.8 eV, related to Ca 2p₃/₂ and Ca 2p₁/₂, respectively [61], while the high-resolution spectra of P 2p showed only a major peak at ∼133.5 eV due to the small spin-orbit splitting [62]. The corresponding oxygen peak was observed at ∼531.4 eV. The peak at ∼284.6 eV corresponds to C 1 s. Ti 2p peaks are observed in the survey spectra of HAT, FHAT, and FAT, whereas no titanium signal is detected in the pure HA coating. The high-resolution spectra of Ti 2p show two peaks at ∼458.4 eV and ∼464.4 eV corresponding to Ti 2p₃/₂ and Ti 2p₁/₂, respectively [63, 64]. A peak at ∼684.4 eV corresponding to F 1 s is observed in FHAT and FAT coatings [8]. The measured Ca/P ratio in the HA, HAT, FHAT, and FAT coatings was 1.66, 1.64, 1.63, and 1.61, respectively.

**Fig. 4:**
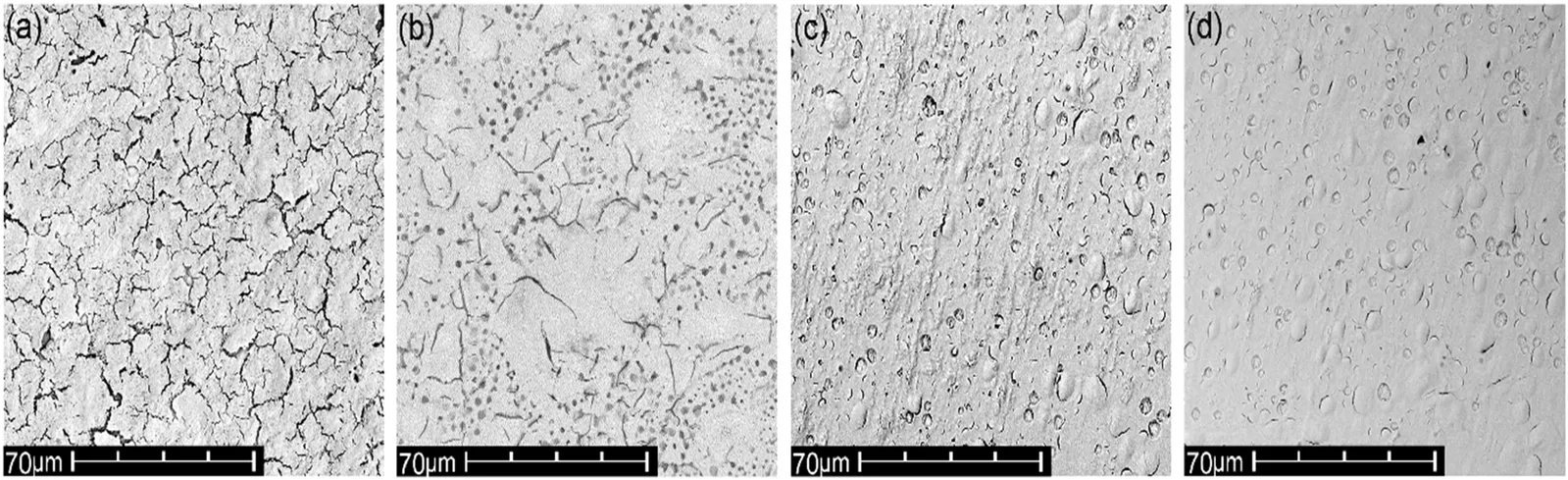
SEM micrographs of the surface of the deposited coatings: **a** HA; **b** HAT; **c** FHAT; and **d** FAT

The SEM micrographs for the surface morphologies of the developed coatings on Ti6Al4V are presented in Fig. 4. The surface morphological differences between HA and the other composite coatings can be observed. Micro-cracks were observed in the structure of the HA layer. The presence of CaTiO₃ in HA-based coatings resulted in fewer surface defects, with a distribution of discontinuous and limited micro-cracks compared to the pure HA layer. The FHAT and FAT coatings show similar surface morphologies, characterized by a further reduction of structural micro-cracks compared to the HAT coating.

**Fig. 5:**
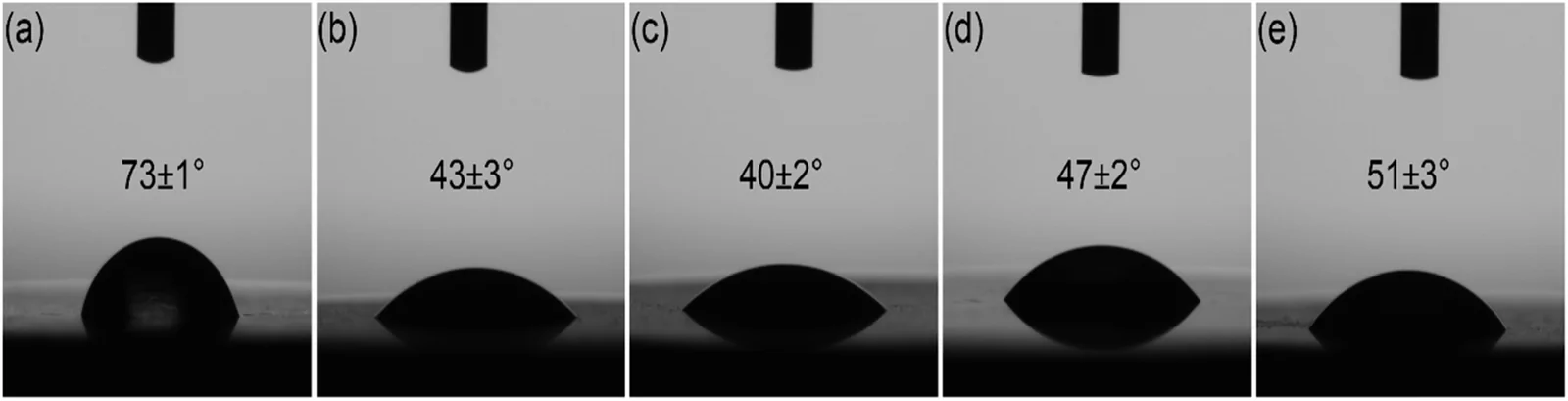
Contact angle measurement: **a** Ti6Al4V alloy; **b** HA; **c** HAT; **d** FHAT; and **e** FAT

The wetting behavior of the uncoated and coated Ti6Al4V alloy was studied by measuring the contact angle of SBF sessile drops, as shown in Fig. 5. The uncoated Ti6Al4V alloy exhibited a half-spherical drop shape, indicating poor hydrophilic quality. The HA- and HAT-coated samples exhibited significantly lower contact angles compared to the uncoated alloy. Upon fluoride incorporation into the HAT structure, the contact angle increased for both FHAT and FAT coatings.

**Fig. 6:**
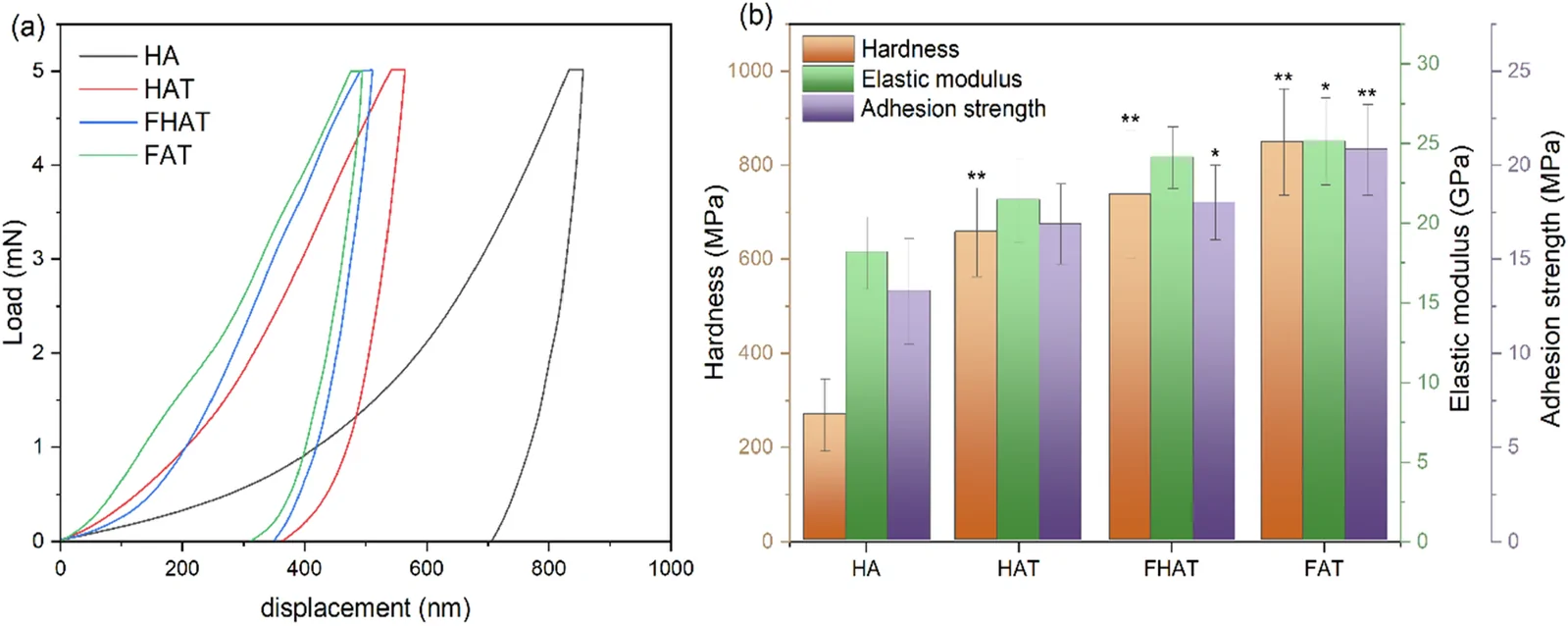
**a** Nanoindentation plot; **b** Hardness, elastic modulus, and adhesion strength of coating layers on Ti6Al4V substrates. Statistically, significant differences were indicated as *p < 0.05 and **p < 0.01 compared with HA

Nanoindentation and pull-out tests were carried out to assess the mechanical properties of the applied coatings. It becomes clear from the indentation curves (Fig. 6a) that composite coatings have higher hardness than the pure HA coating. The values of adhesion strength, elastic modulus (E), and hardness (H), calculated using the Oliver–Pharr method, are shown in Fig. 6b [53]. The hardness and elastic modulus of the HA coating were measured as 269 ± 77 MPa and 18.1 ± 2.3 GPa, respectively. These values are much lower than those of bulk pore-free HA (H = 9.9 ± 0.5 GPa and E = 110 ± 4 GPa [65]). The incorporation of CaTiO₃ into the HA matrix resulted in a clear improvement in the mechanical performance of the coatings, with the HAT composite coating exhibiting higher hardness and elastic modulus (657 ± 94 MPa and 21.6 ± 2.6 GPa, respectively) compared to pure HA. Upon fluoridation of the composite coating, the hardness and elastic modulus were further improved for FHAT (H = 738 ± 135 MPa and E = 24.1 ± 1.9 GPa) and FAT (H = 849 ± 113 MPa and E = 25.1 ± 2.7 GPa) coatings.

The H/E ratio improved from 0.0148 for pure HA coatings to 0.0306 for HAT, 0.0306 for FHAT, and 0.0338 for FAT coatings.

A pull-out test was carried out to evaluate the adhesion strength of the applied coating layers, and the results and statistical differences between the studied groups are shown in Fig. 6b. The HA and HAT coatings exhibited mean adhesion strength values of 13.3 ± 2.8 MPa and 16.8 ± 2.1 MPa, respectively; however, this difference was not statistically significant (*p* > 0.05). Fluoridation of the HAT composite further enhanced the adhesion strength, which significantly increased to 18 ± 2 MPa for the FHAT coating (*p* < 0.05) and to 20.8 ± 2.4 MPa for the FAT coating (*p* < 0.01).

**Fig. 7:**
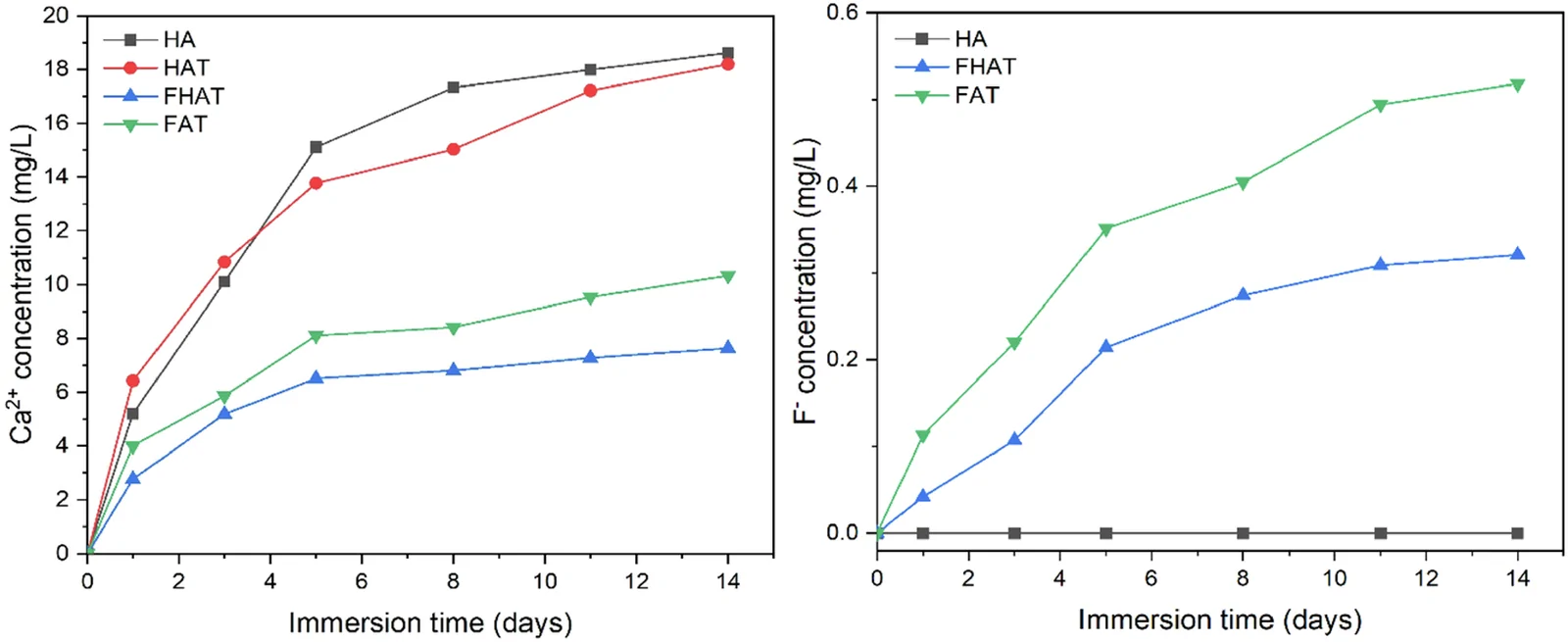
Dissolution profiles of composite coatings as a function of immersion time

The dissolution behavior of the composite coatings, based on the release of Ca²⁺ and F⁻ ions in PBS solution, is presented in Fig. 7 as a function of immersion time. The release of Ca²⁺ ions increased with increasing immersion time for all tested coatings. The pure HA and HAT composite coatings showed a comparable and relatively high dissolution rate. Upon fluoridation of the composite coating, the dissolution rate was noticeably reduced. However, the composite coatings did not show a steady reduction of dissolution rate as the fluoridation degree increased; instead, the FAT layer released higher Ca²⁺ ions than the FHAT layer, while still remaining remarkably lower than the pure HA coating.

Fluoride ions in the FHAT and FAT coatings were released within a short period (1 day) but in small concentrations. More F⁻ ions were released with extended immersion time, reaching up to 0.321 mg/L for the FHAT coating and 0.518 mg/L for the FAT coating.

**Fig. 8:**
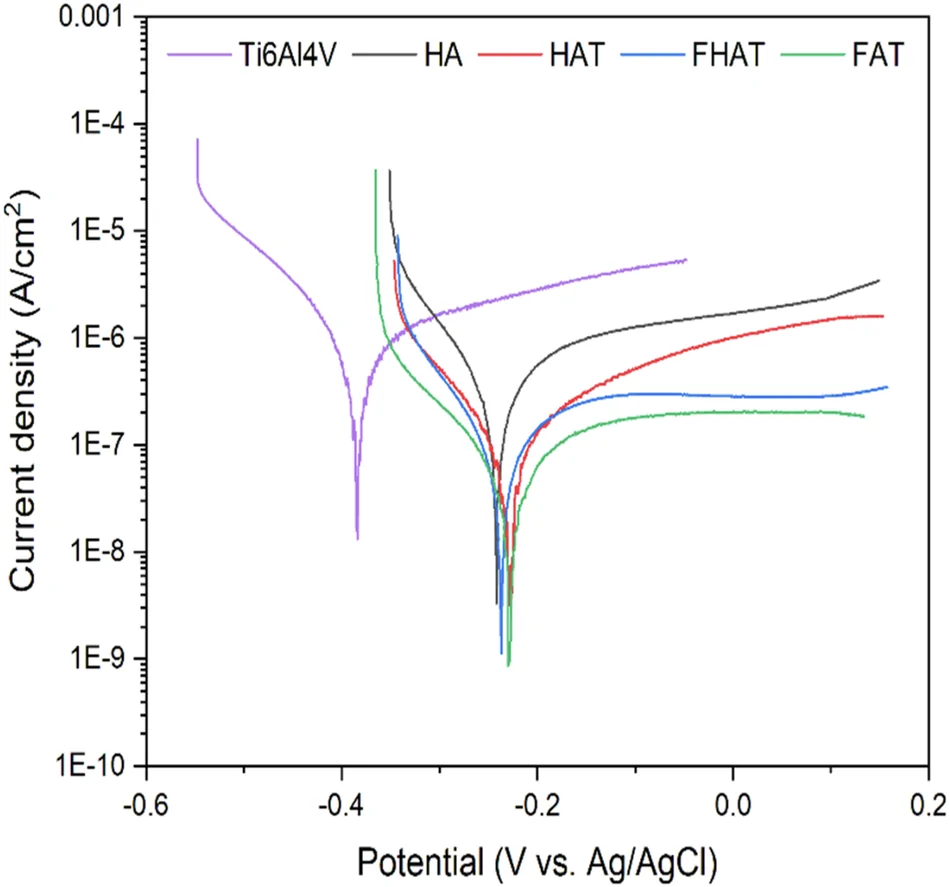
Potentiodynamic polarization curves of uncoated and coated Ti6Al4V alloy in SBF solution

The corrosion behavior of the developed coatings on the Ti6Al4V substrate in SBF was studied by potentiodynamic polarization. The polarization curves and the extracted Ecorr, Icorr, and CR values of the bare and coated Ti6Al4V samples are provided in Fig. 8 and Table 3, respectively. A comparison of the polarization curves shows that the uncoated Ti6Al4V has the highest susceptibility to corrosion. Applying the HA coating noticeably decreased the Icorr and shifted Ecorr to a nobler value. The CaTiO₃-containing HAT coating showed a further reduction in Icorr compared to the HA coating. The corrosion potential of the HAT coating ( − 0.2281 V vs. Ag/AgCl) was increased compared to the pure HA coating ( − 0.2416 V vs. Ag/AgCl).

**Table 3:** Electrochemical parameters extracted from polarization curves

Parameter	Ti6Al4V	HA	HAT	FHAT	FAT
*E*_corr_ (V vs. Ag/AgCl)	−0.384	−0.242	−0.228	−0.237	−0.229
*I*_corr_ (A cm^−2^)	90.4 × 10^–8^	29.5 × 10^–8^	9.57 × 10^–8^	8.54 × 10^–8^	5.15 × 10^–8^
CR (mm/y)	20.7 × 10^–3^	6.75 × 10^–3^	2.19 × 10^–3^	1.95 × 10^–3^	1.18 × 10^–3^

Fluoridation of the composite coatings further improved the electrochemical behavior of the coated Ti6Al4V alloy. The corrosion potential of the FHAT coating shifted slightly toward a negative value, while the FAT coating shifted to more positive values with respect to the HAT coating. The corrosion current density decreased from 9.57 × 10⁻⁸ A/cm² for HAT to 8.54 × 10⁻⁸ A/cm² for FHAT and 5.15 × 10⁻⁸ A/cm² for FAT.

**Fig. 9:**
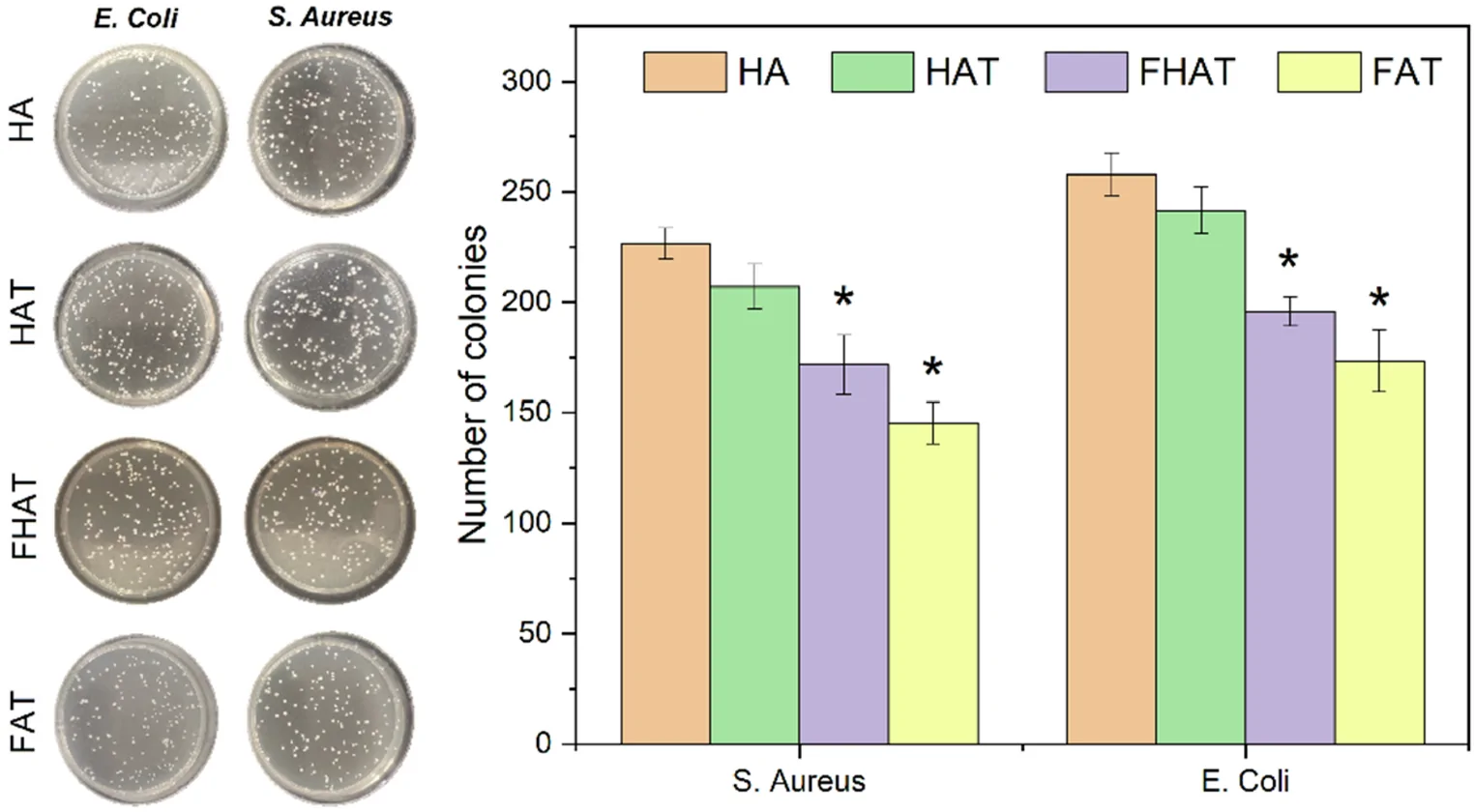
Antibacterial efficacy of the coatings against *E. coli* and *S. aureus* bacteria. Values represent the mean ± standard deviation of triplicate experiments. Statistically, significant differences were indicated as *p < 0.05 compared with HA

The potential antibacterial efficacy of the developed coatings was investigated against S. aureus and E. coli, the most common infection-causing microorganisms. The plate-counting method evaluated the antibacterial activity of HA, HAT, FHAT, and FAT coatings against the studied bacteria, and the number of surviving colonies is shown in Fig. 9. The HAT composite coating showed a slight antibacterial effect; the number of E. coli and S. aureus colonies was inhibited by approximately 3 and 10%, respectively. The incorporation of fluoride improved the inhibition of bacterial growth; the amounts of E. coli and S. Aureus colonies were reduced by approximately 28 and 31% for the FHAT coating and 32 and 33% for the FAT coating, respectively.

Based on the antibacterial assays, Gram-positive S. aureus bacteria showed greater susceptibility to the studied coatings than Gram-negative E. coli.

**Fig. 10:**
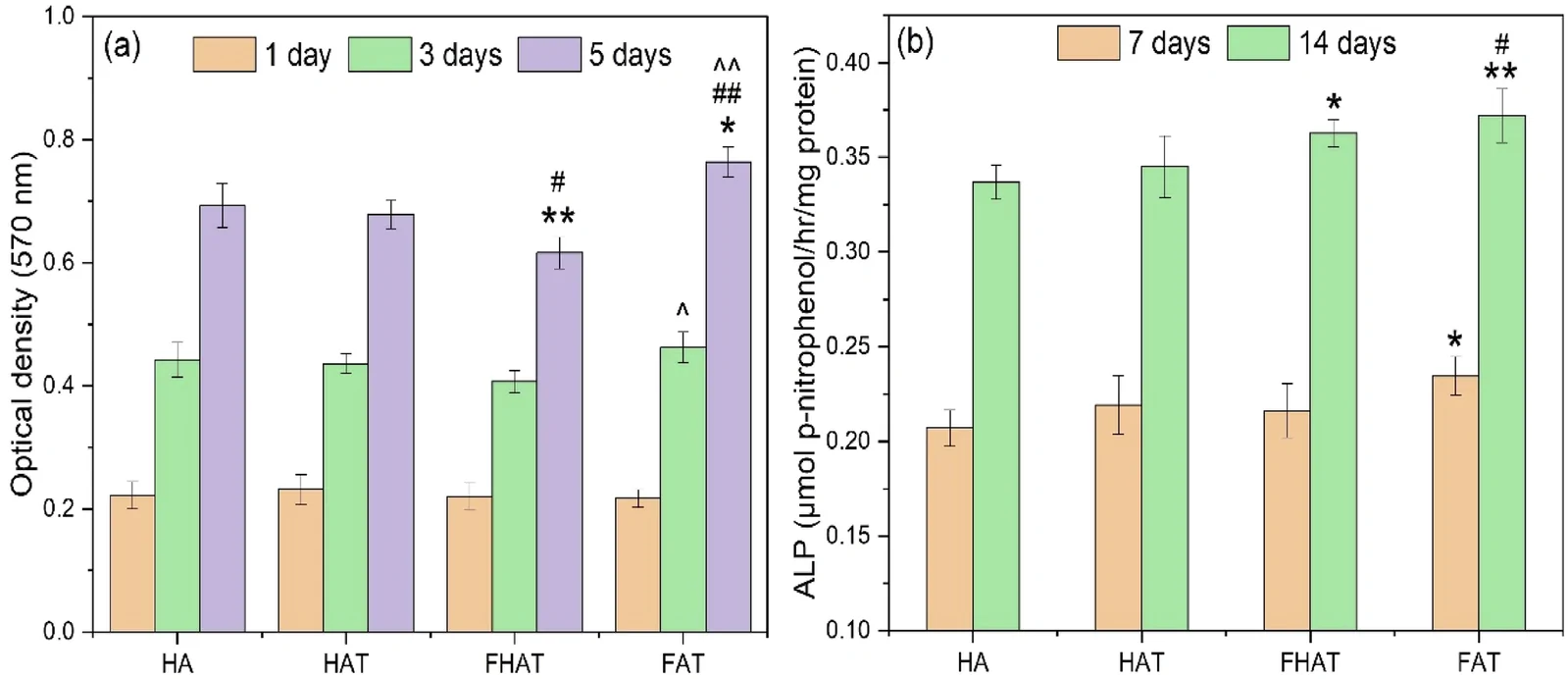
Cell proliferation (**a**) and ALP activity (**b**) of MG-63 cells upon exposure to the studied coatings. Statistically significant differences were indicated as follows: *p < 0.05, **p < 0.01 compared with HA, ^#^*p* < 0.05, ^##^*p* < 0.01 compared with HAT and ^*p* < 0.05, ^^*p* < 0.01 compared with FHAT

The proliferation of MG-63 osteoblastic cells cultured on HA, HAT, FHAT, and FAT coatings was evaluated using the MTT assay at different time intervals, as shown in Fig. 10a. Cell proliferation increased over time for all coatings. After 1 day of culture, no significant differences were observed among any of the samples (*p* > 0.05). After 3 and 5 days of culture, the presence of CaTiO₃ in the HAT composite did not lead to statistically significant changes in proliferation compared to pure HA (*p* > 0.05). The cell proliferation on the FHAT coating was slightly lower than on the HA and HAT coatings. The cell viability on the FAT coatings was significantly higher than that of pure HA and FHAT.

Osteogenic differentiation of MG-63 osteoblastic cells on the coated samples was assessed by quantifying ALP activity at 1 and 2 weeks, as shown in Fig. 10b. The amount of ALP produced increased with culturing time for all studied coatings. The pure HA coating showed the lowest ALP activity in the first week compared to the other composite coatings. The ALP activity on the FAT-coated samples was significantly higher than on pure HA-coated samples at week 1 (*p* < 0.05), while no significant differences were observed among the other composite coatings. After more prolonged incubation, HA and HAT showed similar cellular responses. The ALP activities on the fluoridated composite coatings were significantly higher compared to the undoped coatings at week 2 (*p* < 0.01).

## Discussion

The present study investigates the combined effect of CaTiO₃ incorporation and fluoride substitution in sol–gel-derived hydroxyapatite coatings on Ti6Al4V substrates. While both CaTiO₃-containing HA composites and fluoridated apatite coatings have been individually reported in the literature, their simultaneous integration within a single coating system and the resulting synergistic effects on structural integrity, dissolution behavior, corrosion resistance, antibacterial performance, and biological response remain insufficiently explored. The following discussion interprets the obtained results in the context of these combined modifications and compares them with previously reported findings.

The structural changes observed in the fluoridated samples can be attributed to the substitution of OH groups by fluorine. Replacement of the OH groups in Ca10(PO4)6(OH)2 by fluorine, which leads to the formation of Ca10(PO4)6(OH)2 xFx, reduces the lattice parameter a, while the lattice parameter c remains almost unchanged [58, 66, 67]. The larger crystallite size and lower microstrain observed in FHAT compared to HAT suggest a more ordered crystal lattice upon partial fluoride substitution. The reduction in crystallite size and slight increase in microstrain at higher fluoridation (FAT) reflect changes in defect density and crystallization behavior.

Minor secondary phases detected in the apatite samples (summarized in Table 2) originate from reactions that occurred during the sol–gel synthesis. CaO and Ca(OH)₂ form during the sol–gel processing and thermal treatment [68–70]. CaCO₃ is the product of the reaction of CaO with atmospheric CO₂ during drying and calcination [71]. Ca₂P₂O₇ can arise from thermal decomposition or dehydration of hydrogen phosphate species during heat treatment [72].

The formation of CaTiO₃ is facilitated by mixing the HA, FHA, or FA sol with the Ti-based sol, resulting in a fine-scale distribution of calcium- and titanium-bearing species throughout the hybrid network. During subsequent heat treatment, the Ca–P gel decomposes and produces CaO, while the titanium alkoxide forms Ti–O species that develop into TiO₂. At 600 °C, CaO and TiO₂ react to form CaTiO₃. Similar solid-state reactions between CaO and TiO₂ at relatively low temperatures have been reported in sol–gel Ca–Ti oxide systems, where CaTiO₃ develops upon calcination in the range of approximately 500–700 °C [73–75].

The FTIR spectra provide further evidence of the compositional and structural modifications induced by fluorine incorporation and CaTiO₃ addition. The presence of the low-intensity peak at 875 cm⁻¹ and the band at 1384 cm⁻¹, assigned to the C–O bond of CO₃²⁻ ions, indicates that carbonate substitution had occurred, forming carbonated hydroxyapatite. The peak at 876 cm⁻¹ could also be attributed to the stretch vibration of the P-OH linkage, indicating the presence of HPO₄²⁻ species [76, 77]. The characteristic PO₄³⁻ and OH⁻ vibrational modes of apatite in the HAT spectrum are not significantly altered by the presence of the CaTiO₃ secondary phase. The broad band of Ti-O and Ti-O-Ti radicals associated with CaTiO₃ could overlap with the phosphate bands at 572 and 602 cm⁻¹, as similarly indicated in previous studies [10, 78].

The shift of the OH⁻ band from 3573 cm⁻¹ to 3540 cm⁻¹ (FHAT) and 3537 cm⁻¹ (FAT), the reduction in its intensity, and the absence of the hydroxyl band at 632 cm⁻¹ in the fluoridated samples suggest that hydroxyl groups in the HA structure were successfully substituted by fluoride ions. The additional band at 746 cm⁻¹ observed in the FHAT and FAT spectra might signify the hydroxyl group’s appearance as an OH–F bond [79]. Wei et al. [80] similarly attributed a band at 746 cm⁻¹ to the presence of fluoride ions. However, this band might also be attributed to the P-O-P bridge bond in the apatite spectra, as suggested by Knubovets [81].

The XPS analysis is consistent with the phase composition identified by XRD and FTIR. The Ca 2p and P 2p peaks confirm the presence of Ca and P groups characterizing the apatite structure. The oxygen peak at ∼531.4 eV is attributed to the phosphate and hydroxyl bonds within the apatite lattice and is characteristic of oxygen bonds in organic species [82–84]. The C 1 s peak at ∼284.6 eV is attributed to residual carbon contaminants [85, 86]. The Ti 2p₃/₂ and Ti 2p₁/₂ binding energies at ∼458.4 eV and ∼464.4 eV are typical for Ti⁴⁺ in oxidic environments [63, 64], and based on the XRD results, this Ti⁴⁺ signal is attributed to the CaTiO₃ phase formed during heat treatment of the Ti-containing sols. The F 1 s peak at ∼684.4 eV confirms that fluorine was successfully incorporated into the apatite structure [8].

Even though the ideal Ca/P ratio for stoichiometric HA is 1.67, stable HA phases have been found to exist over a range of Ca/P ratios from 1.3 to 1.8 [87]. The measured Ca/P ratios across all coatings are therefore close to the stoichiometric value and within the range of the stable apatite phase, confirming that the developed composites represent a proper hybrid coating. The results obtained from XPS stand in good agreement with those obtained from XRD and FTIR analysis.

The micro-cracks observed in the pure HA coating can be attributed to the mismatch in coefficients of thermal expansion (CTE) between Ti6Al4V (8.9 × 10⁻⁶ K⁻¹) and HA (15 × 10⁻⁶ K⁻¹). During the sintering process, this mismatch causes residual tensile stress on the HA layer, resulting in the development of a cracked structure. The reduction in surface defects observed in the CaTiO₃-containing coatings suggests that CaTiO₃ may contribute to improved coating integrity and reduced crack propagation within the HA matrix. The further reduction of micro-cracks in the FHAT and FAT coatings can be primarily attributed to the reduced CTE of fluoridated apatite, which decreases to approximately 9.1 × 10⁻⁶ K⁻¹ for fluorapatite, closely matching that of the Ti6Al4V substrate [88–90]. This improved CTE compatibility reduces residual thermal stresses during cooling, thereby suppressing crack formation.

The wettability of the coating surface strongly affects the implant response in the human body [91, 92], as adsorption of body fluid on the surface of coated implants represents the initial stage for cell attachment and proliferation [93]. Generally, a larger contact angle (hydrophobic, ∼90°) limits cell attachment to the coating surface, while superhydrophilic surfaces with extremely low contact angles (<10°) hinder osteoblast cell proliferation. Consequently, a hydrophilic coating surface—neither hydrophobic nor superhydrophilic—has been reported as ideal for cell attachment and proliferation [94, 95].

The significantly lower contact angles observed in the HA and HAT coatings indicate an improved hydrophilic nature and better wetting behavior toward SBF solution, attributed to the OH⁻ groups present in the apatite structure. The negative hydroxyl groups interact with SBF molecules via hydrogen bonds, resulting in lower liquid-solid interfacial energy and enhanced surface hydrophilicity [96]. Incorporating fluoride into the HAT structure reduces the number of exposed hydroxyl groups on the coating surface, which accounts for the increased contact angle observed in FHAT and FAT. Similar outcomes have been reported elsewhere [28, 97].

Generally, the mechanical properties of bioactive coatings play a crucial role in maintaining an appropriate connection with cells. Poor mechanical properties of the coated implant surface can lead to cracks in the coating layer, subsequently causing cell detachment due to cell proliferation-induced stress [98]. The hardness and elastic modulus measured for the pure HA coating, while considerably lower than those of bulk pore-free HA, are consistent with previous research for thin-film coatings [99]. This reduction is attributed to surface defects in the HA coating, such as cracks and porosity observed in the SEM analysis.

The clear improvement in hardness and elastic modulus upon incorporation of CaTiO₃ into the HA matrix can be attributed to the higher stiffness of CaTiO₃, which acts as a mechanically stable secondary phase within the HA matrix. Previous studies on HA/CaTiO₃ systems have reported improved fracture toughness, reduced brittleness, and increased stiffness due to the dispersion of CaTiO₃ particles within the apatite matrix, helping to restrict crack propagation and enhance load-bearing capacity [100, 101]. More recently, Nadian et al. [102] confirmed CaTiO₃ formation in plasma-sprayed HA-Ti composite coatings on Ti6Al4V and reported a substantial increase in adhesion strength from 7.1 MPa for pure HA to 25.2 MPa for the composite, further supporting the beneficial role of CaTiO₃ in enhancing both mechanical performance and interfacial bonding of HA-based coatings. Thus, the formation of CaTiO₃ in the present coatings contributes significantly to the observed mechanical reinforcement. The further improvements in hardness and elastic modulus observed in the fluoridated composite coatings are associated with improved coating morphology and integrity, as well as with the presence of CaTiO₃ secondary phases. SEM observations revealed that fluoridation reduced the formation of micro-cracks and produced a more uniform coating surface, which reduces the tendency for local stress concentration and crack initiation during indentation, consistent with the higher hardness and elastic modulus measured for the fluoridated composite coatings. These findings align well with previous reports on fluoridated apatite coatings [103, 104].

The H/E ratio has been widely used to understand the elastic behavior of material surfaces [105]. Higher H/E values indicate a greater ability of a material to sustain elastic deformation before yielding, which is commonly associated with improved resistance to abrasive wear and reduced plastic damage during mechanical loading [106]. In addition, higher H/E values correlate with greater wear resistance and durability, reducing material degradation in physiological environments [98]. The improvement in H/E ratio from pure HA to the composite and fluoridated coatings is consistent with the mechanical reinforcement role of CaTiO₃ discussed above. The slightly higher H/E ratio observed for FAT coatings may also be related to the reduction of residual tensile stresses in the fluoridated apatite lattice, as fluoride incorporation is known to increase crystallinity and structural stability [107, 108]. Overall, the improved H/E ratios confirm that the fluoridated HA/CaTiO₃ composite coatings exhibit superior elastic performance and mechanical durability compared to pure HA.

The moderate increase in adhesion strength from HA to HAT, while not statistically significant (*p* > 0.05), suggests a beneficial but limited effect of CaTiO₃ on interfacial bonding. The significant enhancement in adhesion strength upon fluoridation could be related to chemical bond formation at the coating–substrate interface during the sintering stage. Since the Ti6Al4V samples have a comparable surface finish, mechanical interlocking is considered identical across all coatings, and the observed improvement is therefore primarily attributed to chemical bonding. Zhang et al. [8] indicated that the abundant F, O, Ca, and P elements in the interface region can form complex chemical bonds with Ti at elevated temperatures, leading to greater adhesion strength. In addition, fluoridation of apatite reduces the CTE difference between the substrate and coating, as discussed above, which reduces residual stresses in the interface region and subsequently improves adhesion strength [109]. Previous studies by Zhang et al. [103] and Tredwin et al. [110] have similarly reported improved adhesion strength of fluoridated apatite compared to pure HA coatings. Ahmadi et al. [105] reported comparable adhesion strength values of up to 20.5 MPa for sol–gel-derived HAp/TiO₂/Ag nanocomposite coatings on Ti6Al4V, consistent with the adhesion strength achieved by the FAT coating in the present study, and further confirming that sol–gel-derived composite coatings on Ti6Al4V can meet clinically relevant bonding requirements.

Dissolution behavior is a crucial parameter in determining the stability and bioactivity of hydroxyapatite coatings in the physiological environment. Releasing Ca²⁺ ions via an appropriate dissolution rate is essential to improve bone formation on the implant by precipitation of an apatite layer on the coating surface and activating the response of osteoblast cells [90]. However, rapid dissolution of the coating could release excessive amounts of Ca²⁺ ions that could cause cytotoxic effects on living cells [111]. In addition, a high dissolution rate deteriorates the mechanical properties of the coating and may loosen the bonding between the host bone and the coated implant [112]. Thus, controlling the dissolution rate of HA coatings is necessary.

The reduced dissolution rate observed upon fluoridation is consistent with the well-established greater dissolution resistance of fluorine-incorporated HA structures against body fluid [26, 113, 114]. The non-monotonic behavior—whereby the FAT layer released more Ca²⁺ than FHAT despite its higher fluoridation degree—could be attributed to the formation of strong hydrogen bonds between fluorine ions and hydroxyl ions when the hydroxyl is only partially substituted by fluorine. The crystal structure of hydroxyapatite is typically characterized by a certain degree of disorder, as the OH⁻ groups adopt an anti-parallel orientation along the b axis and a slight displacement from the symmetrical positions along the c axis [115–118]. Once F⁻ ions are incorporated, a less elastically strained structure is achieved due to their smaller ionic size [119–121]. In addition, F⁻ ions occupy symmetric positions in the apatite structure and reverse the alignment of OH⁻ groups, thereby stabilizing their randomization [122, 123]. Furthermore, the high electronegativity of F⁻ ions enables them to form stronger hydrogen bonds than oxygen. These bonds, formed between F⁻ ions and adjacent OH⁻ groups, contribute to a more stabilized and well-ordered apatite structure, enhancing its chemical stability and resistance [124]. Based on these considerations, the dissolution behavior of apatite coatings is highly sensitive to their chemical composition and fluoridation degree, and could therefore be controlled by replacing hydroxyl groups with fluorine ions according to clinical needs.

The amounts of fluoride ions released from the FHAT and FAT coatings are well below the cytotoxic level ( ~ 10⁻⁵–10⁻⁷ M), falling within the concentration range associated with increased ALP activity and stimulated osteoblastic cell proliferation [125]. The literature further indicates that fluoride ion release from bioglass promotes angiogenic and osteogenic gene expression and collagen formation in osteoblast cultures [126], while Li et al. [27] and Qu and Wei [28] similarly reported stimulated osteoblastic cell activity and improved cell attachment and ALP activity on fluoridated hydroxyapatite. Higher fluoride ion release from the FAT layer compared to FHAT is consistent with its higher fluoride content.

Corrosion of Ti6Al4V implants can induce the release of metal ions into the bloodstream, potentially causing allergic reactions—particularly from Al³⁺ and V⁵⁺ ions—upon contact with surrounding tissues [4]. Moreover, corrosion diminishes the mechanical properties of the implant surface [127]. The corrosion potential Ecorr indicates the thermodynamic tendency of the sample surface to corrode, while the corrosion current density Icorr reflects the kinetics of the corrosion rate. Consequently, higher Ecorr values and lower Icorr values correspond to lower corrosion rates and better anticorrosion properties [128].

The decreased Icorr and nobler Ecorr observed for the HA coating compared to uncoated Ti6Al4V indicate a lower corrosion rate and improved surface stability, consistent with previous reports that HA coatings improve the corrosion behavior of Ti6Al4V alloy by reducing the charge transfer between the substrate and the environment [129]. The further improvement in corrosion resistance of the HAT coating can be attributed primarily to its improved surface morphology, particularly the reduction of micro-cracks observed in the SEM analysis. Micro-cracks act as preferential locations for chloride ions to reach the Ti6Al4V surface; the reduced surface defects in the HAT coating therefore prevent the corrosive SBF solution from penetrating the coating and reaching the substrate. In addition, the slightly higher adhesion strength of the HAT layer contributes to improved interfacial stability under corrosive exposure, further enhancing its resistance to degradation in SBF.

The further reduction in corrosion current density observed for the fluoridated coatings, particularly FAT, demonstrates the positive role of fluorine in enhancing corrosion resistance. This improvement arises from the combined effects of fluoride substitution and the CaTiO₃-containing microstructure, which together promote the formation of more uniform coating layers with fewer micro-cracks. These morphological features hinder the penetration of aggressive ionic species and strengthen the coating–substrate interface. The lower biodegradability of the fluoridated apatite structures, as observed in the dissolution test results, represents an additional factor contributing to the reduced corrosion rate [130]. Zang et al. [131] similarly reported that fluorine-substituted hydroxyapatite coatings on Ti–6Al–4V exhibited reduced corrosion current density and nobler corrosion potential compared to pure HA, attributing this improvement to the stronger electron affinity of fluorine relative to oxygen, which promotes the formation of a more ordered and chemically stable apatite structure, consistent with the corrosion improvement observed for the fluoridated composite coatings in the present study. In summary, the results indicate an efficient passivation ability of the composite coatings that hinders ionic diffusion and offers high corrosion resistance for the Ti6Al4V alloy.

The slight antibacterial effect observed for the HAT coating is too weak to indicate a true antibacterial mechanism and may reflect minor variations in surface morphology that slightly influence initial bacterial adhesion. The more pronounced inhibition observed upon fluoride incorporation is associated with an interaction between the coating surface and bacterial cells as a consequence of F⁻ ion release into the environment, with the FAT coating exhibiting higher fluoride ion release than FHAT. It is suggested that the released F⁻ ions interfere with the metabolic systems of bacterial cells by inhibiting enolase, urease, and catalase enzymes, leading to their death [132]. The antibacterial effect of fluoride dopants, alone or in combination with other antibacterial agents, against various types of bacteria has been demonstrated in the literature. Ge et al. [133] reported active bacterial growth inhibition of fluorhydroxyapatite coating against P. gingivalis, E. coli, and S. aureus. Stanić et al. [132] detected a remarkable reduction of viable bacterial cells of fluorapatite against M. luteus, S. aureus, and K. pneumoniae. Syed et al. [134] indicated that fluoride ions in lithium fluoride compounds exhibit synergistic effects that reduce the minimal inhibitory concentration (MIC) of various antibiotics toward different types of bacteria, including S. aureus and E. coli.

The greater susceptibility of Gram-positive S. aureus compared to Gram-negative E. coli is consistent with observations reported by Ge et al. [133], who similarly found a lower antibacterial effect of fluoridated hydroxyapatite coatings against E. coli than against S. aureus. This behavior is related to the structural differences between Gram-positive and Gram-negative bacteria. Gram-negative bacteria possess a complex cell wall comprising a thin peptidoglycan layer and two cell membranes—an outer membrane and a plasma membrane—while Gram-positive bacteria have a relatively thick cell wall composed of only a plasma membrane and multiple layers of peptidoglycan. The outer membrane of Gram-negative bacteria reduces the permeability of molecules, making them less susceptible to many antibiotic substances than Gram-positive bacteria [135]. It is worth noting that coatings incorporating dedicated antibacterial agents such as silver can achieve substantially higher bacterial inhibition rates; Ahmadi et al. [105] reported 98.9% inhibition of E. coli for a sol–gel-derived HAp/TiO₂/Ag nanocomposite coating on Ti6Al4V. The moderate inhibition rates observed in the present study are therefore consistent with the comparatively mild antibacterial nature of fluoride ions relative to silver-based dopants.

Despite the good antibacterial effect of the fluoridated HA-CaTiO₃ coatings, it was necessary to assess whether the CaTiO₃ and F⁻ content induce cytotoxic effects on human cells. The absence of statistically significant differences in cell proliferation between the HAT composite and pure HA at all time points indicates that the CaTiO₃-containing composite coating is non-cytotoxic. Similar non-cytotoxic behavior of HA/CaTiO₃ composites has been reported by Dubey et al. [18], who observed good cell viability and proliferation on CaTiO₃-containing HA bioceramics.

The slightly lower cell proliferation observed on the FHAT coating compared to HA and HAT suggests a mild suppression of cell activity, which can be attributed to the lower dissolution rate of fluoridated apatite. Coatings that dissolve more readily release higher concentrations of Ca²⁺ ions, which are known to promote osteoblastic proliferation [90, 136]. The higher cell viability observed on FAT coatings compared to both pure HA and FHAT suggests that the release of F⁻ ions positively affects MG-63 cells and further confirms that the proliferation reduction observed for FHAT is unrelated to F⁻ ion release. Overall, incorporating fluoride into the HAT composite structure does not induce cytotoxic effects on MG-63 cells. Wang et al. indicated that fluoridation of CaP at a substitution level similar to that of the present study has no adverse effect on human MG-63 cells. Lee et al. [90] reported comparable cell proliferation of fluorapatite to pure HA coatings, consistent with the present results. Borkowski et al. [79] observed a noticeable improvement in cell viability of fluorapatite coatings compared to pure HA at various sintering temperatures, highlighting the positive role of released F⁻ ions in stimulating osteoblastic cell activity. In vivo studies by Overgaard et al. [137] and Dhert et al. [138] have similarly demonstrated outstanding biocompatibility compared to pure HA. Based on these findings, incorporating fluoride into the HA structure within the conducted experimental domain did not induce adverse cellular responses and demonstrated good in vitro biocompatibility.

The increasing ALP activity over time across all coatings confirms good osteogenic differentiation of MG-63 cells on all studied surfaces. The significantly higher ALP activity observed on fluoridated composite coatings compared to undoped coatings after prolonged incubation (*p* < 0.01) indicates that released fluoride ions stimulate and induce MG-63 cell osteogenic differentiation. These results suggest that fluoridated apatite coatings release fluoride ions into the niches of osteogenic cells, stimulating them to enter a later differentiation stage, consistent with previous reports [125, 139].

Considering the overall performance of the studied coatings across all investigated properties, full fluoride substitution appears to represent the more favorable composition, combining good mechanical properties, enhanced corrosion resistance, lower dissolution rate, and improved biological response compared to partial fluoride substitution. Nevertheless, the slightly reduced cell proliferation observed for the partially fluoridated composite coating suggests that the biological response is sensitive to the degree of fluoride substitution, and that substitution levels beyond those investigated here could risk shifting fluoride release into concentration ranges with adverse cellular effects. The substitution range examined in this study therefore defines a biologically safe and practically relevant processing window for fluoridated HA/CaTiO₃ composite coatings on titanium implants.

## Conclusions

In this work, sol–gel-derived hydroxyapatite/CaTiO₃ and fluoridated hydroxyapatite/CaTiO₃ composite coatings were successfully applied onto Ti6Al4V substrates, and their structural, mechanical, chemical, and biological responses were systematically investigated for CaTiO₃-containing coatings as a function of fluoride incorporation and compared to pure HA coatings.

The incorporation of CaTiO₃ into the hydroxyapatite matrix (HAT) resulted in a clear improvement in the coating integrity and mechanical performance, as reflected by increased hardness, elastic modulus, and H/E ratio, together with enhanced corrosion resistance. These improvements are attributed to the presence of a mechanically stable CaTiO₃ secondary phase, which contributes to improved load transfer and coating cohesion.

Subsequent fluoridation of the CaTiO₃-containing coatings (FHAT and FAT) reduced the distortion of the crystal lattice of hydroxyapatite and the formation of surface micro-cracks. Furthermore, it improved adhesion strength and significantly lowered dissolution and corrosion rates in SBF, while maintaining fluoride release well below cytotoxic levels. At the same time, fluoridated composite coatings—particularly FAT—exhibited enhanced antibacterial activity against E. coli and S. aureus, along with increased MG-63 cell proliferation and osteogenic differentiation compared to pure hydroxyapatite.

These findings indicate that fluoridated HA/CaTiO₃ composite coatings offer a more favorable combination of mechanical integrity, chemical stability and biofunctionality than conventional HA coatings. Beyond confirming individual effects of CaTiO₃ or fluoride reported previously, the present study defines a compositional and processing window in which simultaneous CaTiO₃ formation and controlled fluoridation can be used to tailor the degradation behavior, corrosion resistance, and biological performance of apatite coatings on titanium implants for long-term load-bearing applications.

Nevertheless, the present study has certain limitations that should be acknowledged. The biological evaluation was conducted exclusively in vitro, and the long-term performance of these coatings under physiological loading conditions remains to be validated. Future work should therefore focus on in vivo assessment of the fluoridated HA/CaTiO₃ composite coatings in relevant animal models, as well as investigation of their mechanical durability under cyclic loading conditions representative of load-bearing implant applications. Future work should also include quantitative surface roughness measurements to further correlate surface topography with cellular responses.

## Supplementary information

Supplementary information

## Data Availability

The data presented in this study are available in the article and the supplementary information.
