# Supplementary material for: Development of sol–gel derived fluoridated hydroxyapatite/CaTiO3 composite coating on titanium implant
Source: J Mater Sci Mater Med. 2026 Nov 13;37(1):137. doi: 10.1007/s10856-026-07142-y (PMC13768235; doi:10.1007/s10856-026-07142-y)
Supplement: Supplementary file 1 [file 10856_2026_7142_MOESM1_ESM.docx]

Development of sol-gel derived fluoridated hydroxyapatite/CaTiO3 composite coating on titanium implant

*Alaa Jaafar^1,2^, Christian Schimpf^3^, Marcel Mandel^4^, Hans Lesny^1^, Christine Hecker^1,2^, David Rafaja^3^,*

*Lutz Krüger^4^, Pal Arki^1,2*^, Yvonne Joseph^1,2^*

*^1^Institute of Nanoscale and Biobased Materials, TU Bergakademie Freiberg, Freiberg, Germany
^2^ Center for Efficient High Temperature Processes and Materials Conversion, TU Bergakademie Freiberg, Freiberg, Germany
^3^ Institute of Materials Science, TU Bergakademie Freiberg, Freiberg, Germany
^4^ Institute of Materials Engineering, TU Bergakademie Freiberg, Freiberg, Germany*

* Corresponding author: [Pal.Arki@esm.tu-freiberg.de](mailto:Pal.Arki@esm.tu-freiberg.de)

Figure S1. High-resolution XPS spectra of the FAT composite coating.
